# Awe reduces depressive symptoms and improves well-being in a randomized-controlled clinical trial

**DOI:** 10.1038/s41598-025-96555-w

**Published:** 2025-05-12

**Authors:** María Monroy, Michael Amster, Jake Eagle, Felicia K. Zerwas, Dacher Keltner, Javier E. López

**Affiliations:** 1 Department of Psychology, Yale University, 100 College St, New Haven, CT 06510 USA; 2 College of Osteopathic Medicine, Touro University California, Vallejo, CA USA; 3 Live Conscious, Hawi, HI USA; 4 Department of Psychology, University of California, Berkeley, CA USA; 5 Center for Reducing Health Disparities, University of California, Davis, 2921 Stockton Blvd, Sacramento, CA 95817 USA; 6 Department of Psychology, New York University, New York, NY USA; 7 Division of Cardiovascular Medicine, Department of Internal Medicine, University of California, Davis, CA USA

**Keywords:** Awe, Stress, Depression, Well-being, Long COVID, Psychology, Signs and symptoms, Quality of life

## Abstract

Emerging evidence suggests that experiences of awe benefit health and well-being. The present investigation examined the efficacy of an awe intervention to improve the psychological health—stress, anxiety, depression, and well-being—of patients living with long COVID. The awe intervention, a Randomized-controlled Clinical Trial, was delivered in synchronous online sessions to patients, across the United States (in April 2023), who met the criteria for long COVID. Results revealed significant improvements in psychological health for those in the awe intervention (*N* = 30), compared to the control group (*N* = 38): including decreased stress, decreased depression symptoms, and increased well-being. There were no significant differences between groups in anxiety symptoms. Effect sizes ranged from medium to large (*d* = 0.78–0.96), demonstrating the robustness of these findings. This work is the first to document that awe can have salutary effects on psychological health, such as reducing symptoms of depression. These findings suggest that a brief awe intervention can improve psychological health in people dealing with chronic stress and physical ailments, as in the case of long COVID.

**Trial registration**. The trial is registered at ClinicalTrials.gov (NCT05676008, 09/01/2023).

**Supplementary Information:**

The online version contains supplementary material available at 10.1038/s41598-025-96555-w.

## Introduction

The nascent science of awe suggests that this emotion, which is often ineffable, has beneficial effects on physical and psychological health^1^. We expand on this literature by examining the efficacy of an awe intervention to improve psychological health—specifically stress, anxiety, depression, and well-being—of patients living with post-acute sequelae after SARS-CoV-2 infection, otherwise known as long COVID.

### Psychological health in the time of COVID-19

The COVID-19 pandemic has proven to be a profound source of mental and physical malaise for people around the world. Evidence suggests that over the course of the pandemic, there was a 25% rise in the prevalence of anxiety and major depression worldwide^2^. This is consistent with other work suggesting that during the acute phase of the pandemic, people in the United States reported worse psychological health, including elevated anxiety, depression, stress, sleeplessness, and loneliness^3,4^.

For some individuals, such as those who are currently suffering from long COVID, the negative effects of the pandemic linger beyond 12 weeks^5,6^ and are likely amplified by this prolonged duration. For example, studies show that people suffering from long COVID report elevated levels of stress, anxiety, and depression^6–9^. Longitudinal evidence of long COVID patients suggests that while physical symptoms such as bodily aches subside over time, psychological health issues such as anxiety and depression persist years after the initial infection^6^. This evidence highlights the need for an intervention that can improve the psychological health of people living with long COVID. In the present work, we developed a brief awe intervention that can be delivered remotely and examined its effects on psychological health outcomes of long COVID patients.

### Awe as a pathway to greater psychological health

The study of positive emotions is a rich area of inquiry revealing numerous benefits for physical and psychological health^10–13^. Within this rich emotion space, recent research has focused on investigating positive emotions as potential targets for intervention^12,14,15^. Evidence is robust and suggests *awe*, the focus of this work, as a promising pathway to greater psychological health outcomes^1,16–18^.

Awe is an emotion elicited by stimuli that are vast, or beyond one’s current perceptual frame of reference^19^. This vastness can be physical, conceptual, or semantic, and requires that extant knowledge structures be accommodated to make sense of what is being perceived^19^. Awe is often experienced through encounters with other people’s courage and kindness, nature, collective gatherings, art, religious and/or spiritual practices, epiphanies, birth and death^1,19–22^.

Empirical studies find experiences of awe to be associated with a range of psychological health benefits^1,17,23,24^. For example, studies have found that daily experiences of awe were associated with lower reports of stress^17,23^. Importantly, these effects remained after controlling for experiences of other positive emotions. In studies in the lab, inductions of awe reduced physiological reactions to stress such as sympathetic autonomic arousal^23^. Awe experiences in nature were found to be related to less rumination^25^ and reduced stress and symptoms of post-traumatic stress disorder in adolescents from under-resourced inner-city schools and combat veterans^24^.

Particularly germane to the present work, a study examining daily awe during the peak of the COVID-19 pandemic in 2020 found that when community adults and healthcare workers experienced more awe, they reported feeling less stress, less physical health symptoms (e.g., bodily pains), and greater well-being^17^. In studies across diverse methodologies—in the lab, daily diaries, and field studies— and across different contexts including the COVID-19 pandemic, awe has robustly predicted improvements in well-being^17,23,24,26,27^. These findings point to awe as a promising intervention for people living with the long-lasting consequences of the COVID-19 pandemic.

## The current research

In the present investigation, we aimed to determine the efficacy of a brief awe intervention on the psychological health of individuals living with long COVID. Long COVID patients can experience negative psychological health—including elevated anxiety and depression— for months and in some cases over a year post COVID infection^5,6^. We add to the existing literature by evaluating, for the first time, the effects of an awe intervention on psychological health outcomes— including stress, anxiety, depression, and well-being— on this unique patient population. We used a randomized clinical trial (RCT) design to evaluate the following hypotheses: People in the awe intervention group, compared to those in the control group, will show significant (1) decreases in stress, (3) decreases in anxiety, (4) decreases in depressive symptoms, and (5) increases in well-being.

## Methods

### Participants

**Fig. 1:**
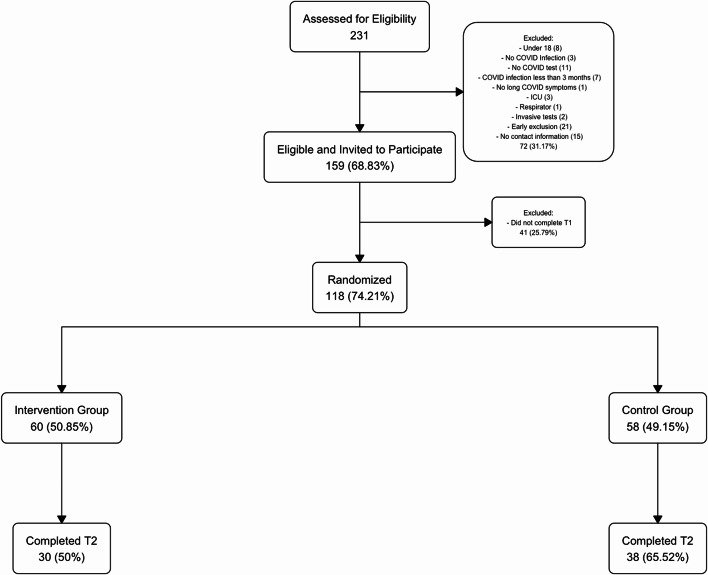
Intervention flowchart. Flowchart shows participant enrollment, randomization, and attrition in the study. See supplement for additional details on exclusion criteria and attrition. T1 denotes baseline/pre-intervention, and T2 denotes post-intervention.

The study design was a single-blind, waitlist-control, RCT. Participants were 68 community members across the USA (*M*_*age*_ = 54.09, *SD* = 13.32, 82.4% female, 14.7% male, 1.5% other, 1.5% NA) who met the Center for Disease Control and Prevention (CDC)^5^ criteria for long COVID (see Fig. 1 for enrollment flowchart). Given the unpredictability of the COVID-19 pandemic, we restricted enrollment of participants to a 3-month remote self-enrollment period and all participants were randomized at one time. Our final sample size, on par with other RCTs for long COVID patients^28^, was calculated to detect an effect size of 0.61 or larger at 80% power. See Table 1 for sample demographics.

**Table 1:** Sample descriptives.

	Awe intervention *N* = 30 %	Control *N* = 38 %
Gender		
Female	76.7	86.8
Male	20	10.5
Other/NA	3.3	2.6
Age *(M*,* SD)*	54.73 (14.69)	53.58 (12.32)
19-29	6.7	2.6
30-39	10	7.9
40-49	26.7	28.9
50-59	16.7	23.7
60-69	23.3	28.9
70+	16.7	7.9
Ethnicity		
Black/African American	3.3	2.6
East Asian/Asian American	6.7	5.3
Latin American	6.7	2.6
White American	73.3	68.4
Other	0	2.6
Mixed ethnicity	10	18.4

Percentages are reported for all variables. For Age, we also report the mean (*M*) and standard deviation (*SD*).

### Procedure

Participants from the general community were recruited in early 2023 for a total of 3 months via flyers at specialty clinics and social media announcements that guided participants to postings on the UC Davis Health StudyPages portal^29^ (see supplemental materials for details). All interested participants provided written informed consent, then took a short eligibility questionnaire via the Qualtrics online platform. Eligibility included the CDC long COVID criteria: (i) an acute COVID infection documented by a positive test more than three months prior to time of recruitment, and (ii) currently having at least one of the most-reported long COVID symptoms (e.g., cough, loss of smell, brain fog, fatigue). See supplemental materials for detailed inclusion and exclusion criteria.

Eligible participants were invited to participate in the study in April 2023, consented to participate, and then asked to complete an entrance online survey prior to scheduling their intervention start date. Participants were informed at the time of consent that their online sessions (i.e., the intervention) would start either within 1 week or > 28 days later to accommodate the large number of participants. After completing the entrance survey, participants were randomized into one of two groups: the intervention group and the waitlist- control group (referred to as ‘control group’ from here forth). Then, they were informed of the start date of their online sessions. The intervention started five days after the entrance survey for the intervention group (Group 1), and an additional four weeks for the control group (Group 2). Per our pre-specified analytic plan, here we focus on data pre and post intervention—four weeks after the entrance survey.

There were four weekly synchronous online sessions in total that were 60 minutes long. The first online session consisted of a presentation on the basics of the study, the science of awe, and a core intervention component that entailed a discussion of how to find awe in daily life (see intervention section). Most information was presented during the first session and reviewed in the following three sessions. All online sessions were presenters’ view only, and no interaction between participants was allowed. Questions for moderators were allowed through a chat box. All sessions were delivered Monday evenings at 5:30pm PDT and participants had unlimited access to video recordings of the sessions for 24 hours. Once the intervention started, for each group irrespective of intervention start date, all participants received a link to online surveys on Fridays at 5pm. Each survey began with Likert-type questions that prompted participants to report on their emotions, thoughts, and experiences during the past week or month. The focus of this report is on data from pre-intervention measures and a month follow-up (post-intervention for Group 1, See Figure S1). See supplemental materials for additional details.

All participants provided written informed consent, and all aspects of the study design and procedure were approved by the UC Davis Institutional Review Board (UCD IRB approval number 1840049-2). The study was conducted in accordance with guidelines and regulations of the UCD IRB and with the Declaration of Helsinki ethical standards. The trial was registered at *ClinicalTrials.gov* (NCT05676008, 09/01/2023).

## Materials and measures

### Awe intervention

The intervention component consisted of teaching participants a simple three-step process of how to find awe in the ordinary: by paying attention to the environment in daily life, slowing down, and expanding on those awe moments. Participants were given the following instructions:

How to access moments of AWE in the ordinary

**A**ttention: full and undivided attention on things you appreciate, value, or find amazing.

**W**ait: slow down, pause.

**E**xhale + Expand: amplify whatever sensations you are experiencing.

Participants were asked to practice finding *awe* at least three times a day. They were also reminded that finding awe does not require extraordinary events and can be practiced in brief moments throughout the day—less than 30 seconds, three times a day.

### Measures

In the pre-intervention questionnaire, participants provided demographic information and reported on their psychological health. All measures, except for demographics, were assessed again post-intervention. An option to decline answering was included in all response scales.

#### Awe

Awe was assessed with a single item in which participants rated the extent to which they felt awe during the past week on a scale from 1 (*not at all*) to 7 (*extremely*)^30^.

#### Positive emotions

Positive emotions were assessed with single items in which participants rated how much of each of nine positive emotions they experienced the past week on scale from 1 (*not at all*) to 7 (*extremely)*: Amusement, Compassion, Contentment, Desire, Elation, Gratitude, Interest, Love, and Pride^30^. To assess overall positive emotionality, all items were aggregated into a composite (α_2_ = 0.83).

#### Stress

Subjective stress was measured with four items of the perceived stress scale (PSS)^31^, in which participants reported how often they felt or thought in certain ways during the last month (four weeks) on a scale from 1 (*never*) to 5 (*very often*): (1) *how often have you felt that you were unable to control the important things in your life? (2) how often have you felt confident about your ability to handle your personal problems?* (3) *how often have you felt that things were going your way?* (4) *how often have you felt difficulties were piling up so high that you could not overcome them?* All items were aggregated into a composite (α_1_ = 0.84, α_2_ = 0.76). See supplemental material (Table S1) for additional details about the stress measure.

#### Anxiety

Anxiety symptoms were assessed with the seven-item generalized anxiety disorder (GAD-7) scale^32^. Participants rated how much they were bothered by certain problems in the past month on a scale from 1 (*not at all*) to 5 (*a great deal*): e.g., *Feeling nervous*,* anxious*,* or on edge; Worrying too much about different things*. All items were aggregated into a composite (α_1_ = 0.92, α_2_ = 0.91).

#### Depression

Depression symptoms were assessed with twelve items from the Beck Depression Inventory (BDI) Short Form^33^. Participants read four statements per question and indicated which statement described the way they had been feeling over the last month on a scale from 0 (e.g., *I do not feel sad*) to 3 (*I am so sad and unhappy that I cannot stand it*). Questions about suicidality were not included due to the nature of remote, asynchronous, self-reporting. A sum score was created of all items (α_1,2_ = 0.83).

#### Well-being

Well-being was measured using the Mental Health Checklist (MHC-SF)^34,35^. The MHC-SF is a well-rounded measure of well-being that includes emotional, psychological, and social well-being. Participants responded to fourteen items indicating the frequency of how they felt during the past month on a scale from 0 (*never*) to 5 (*every day*): e.g., *satisfied with life*; *that [they] had something important to contribute to society*. All items were aggregated into a composite (α_1,2_ = 0.92).

### Data analytic plan

All statistical analyses were performed using RStudio in the R programming environment (version 4.1.2). Our preliminary analyses included examination of missing data and data exclusion based on the planned specified criteria. As part of our preliminary analyses, we also examined differences in adherence and attrition, and the effectiveness of randomization.

For our primary analyses, we used a between-group approach to examine the efficacy of the awe intervention, compared to the control group, in outcomes of interest. To examine the effects of the intervention on psychological health outcomes, we computed changes in psychological health measures from baseline (pre-intervention) to a month follow-up (post-intervention). This type of analysis allows us to examine precise increase or decrease of psychological health assessments during the intervention period. Of note, change and follow-up scores yield similar results. Also, an intention-to-treat analysis was not conducted due to the requirement for complete T2 data from all randomized participants.

## Results

### Preliminary analysis

Table 2 depicts descriptive statistics of pre- and post-intervention measures for both the intervention and the control group. To ensure our random assignment was effective, we first examined differences between the intervention and the control group in baseline/pre-intervention (T1) measures. We found no significant differences in the demographics or pre-intervention measures between the intervention and control group (*p*s ≥ 0.25; see Tables 1 and 2). This suggests that our random assignment was effective and thus we proceeded to examine the effects of the awe intervention. Of note, we also found no significant differences between those who completed the intervention and those who did not (see supplemental materials for additional details).

**Table 2:** Descriptive statistics for T1 and T2 and relative changes of outcome measures.

Outcome measure	Time	Awe intervention		Control	
		M (SE)	% ∆	M (SE)	% ∆
Stress	T1	3.28 (0.15)		3.31 (0.14)	
	T2	2.90 (0.13)	− 12%	3.39 (0.11)	+ 2%
Anxiety	T1	2.95 (0.15)		3.17 (0.18)	
	T2	2.65 (0.16)	− 10%	3.08 (0.16)	− 3%
Depression	T1	12.87 (0.94)		13.00 (0.89)	
	T2	10.62 (0.86)	− 17%	13.21 (0.83)	+ 2%
Well-being	T1	2.55 (0.18)		2.83 (0.16)	
	T2	2.95 (0.17)	+ 16%	2.60 (0.16)	− 8%

T1 denotes baseline/pre-intervention, and T2 denotes post-intervention. *M* denotes the mean and *SE* the standard error. % ∆ denotes percent change from baseline to post-intervention within group, with – or + indicating directionality of change.

### Primary analyses

We first examined the efficacy of the intervention to promote awe. We found that participants in the intervention group (*M*_*Awe*_ = 4.60, *SE* = 0.26) reported experiencing more awe at T2 compared to the control group (*M*_*Control*_ = 2.89, *SE* = 0.29, *t*(64.99) = 4.34, *p* < 0.001; Cohens’ *d* = 1.06). We did not find any differences in experiences of overall positive emotionality (*M*_*Awe*_ = 4.14, *SE* = 0.19; *M*_*Control*_ = 3.76, *SE* = 0.17, *p* = 0.141). These findings suggest that the intervention promoted awe and not overall positive emotionality.

We then tested our primary hypotheses by examining the efficacy of the intervention on psychological health outcomes. To examine whether the awe intervention influenced changes in psychological health measures, we examined changes from baseline (T1) to post intervention (T2). We computed changes (∆), by subtracting responses at T1 from T2. In the examination of stress, we found that participants in the intervention group (∆ *M*_*Awe*_ = -0.38, *SE* = 0.10) compared to those in the control group, showed a reduction in stress responses (∆ *M*_*Control*_ = 0.09, *SE* = 0.10; *t*(65.82) = − 3.34, *p* = 0.001).

Next, we examined whether the awe intervention promoted reductions in anxiety and depression symptoms in these long COVID patients. We found that participants in the intervention group (∆ *M*_*Awe*_ = -2.31, *SE* = 0.58) showed decreases in depression symptoms compared to the control group (∆ *M*_*Control*_ = 0.21, *SE* = 0.55; *t*(62.73) = − 3.15, *p* = 0.002). We did not find any significant differences between the intervention and control group in changes in anxiety symptoms (∆ *M*_*Awe*_ = -0.33, *SE* = 0.14; ∆ *M*_*Control*_ = -0.09, *SE* = 0.11, *p* = 0.178).

**Fig. 2:**
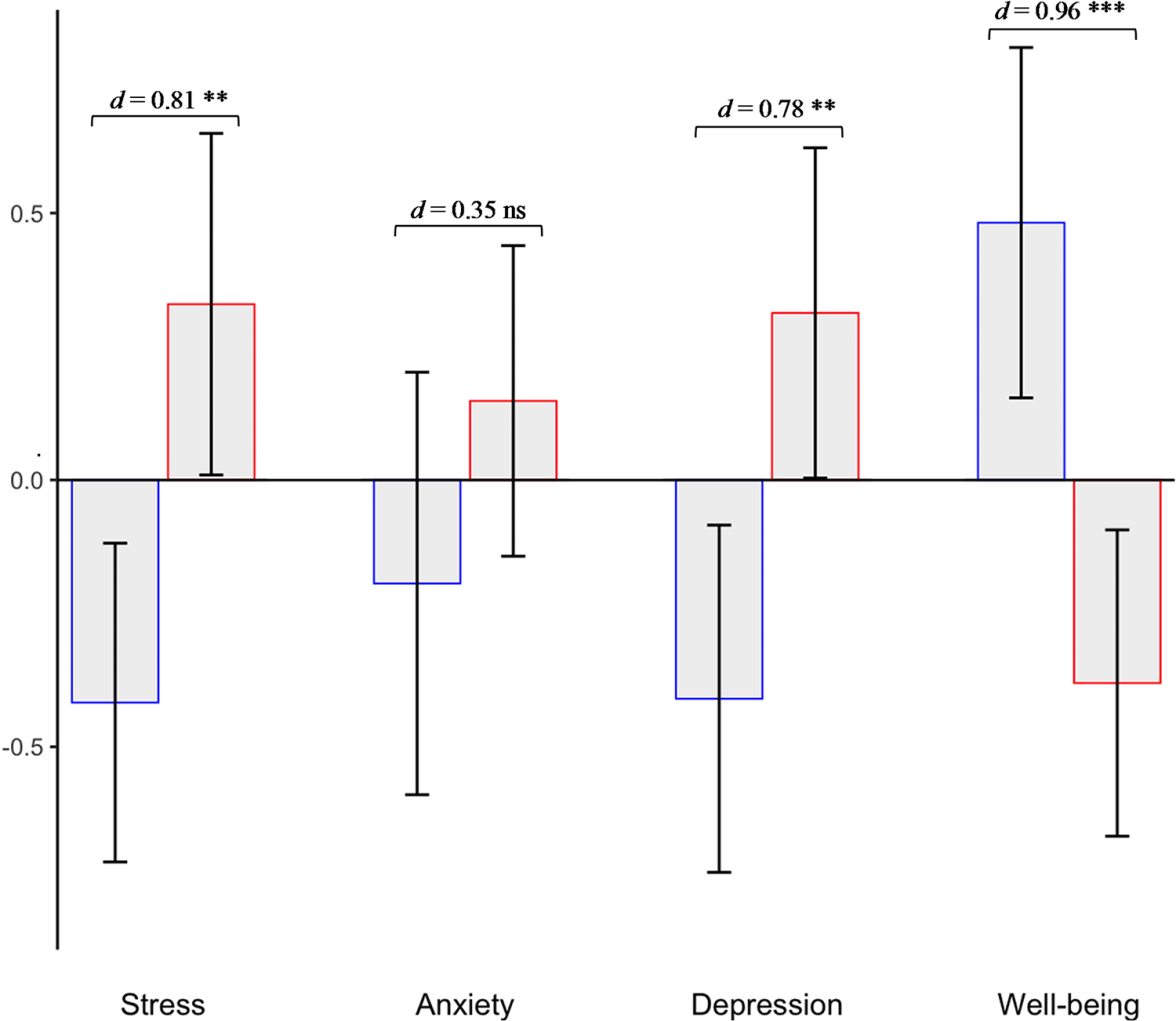
Effects of awe intervention on psychological health. Effect of Awe intervention (first bar; blue) compared to control group (second bar; red) on psychological health outcomes: Stress, Anxiety, Depression, and Well-being. Bars indicate standardized changes from baseline to post intervention, and the error bars indicate 95% confidence intervals. Cohens’ *d* and statistical significance of *t* tests between groups are also reported (ns = not significant, **p < 0.01, ***p < 0.001).

Lastly, we examined the efficacy of the awe intervention to enhance well-being in people suffering from long COVID. We found that participants in the awe intervention (∆ *M*_*Awe*_= 0.40, *SE* = 0.12), compared to the control group, showed increases in well-being (∆ *M*_*Control*_ = -0.22, *SE* = 0.11; *t*(61.91) = 3.88, *p* < 0.001). See Fig. 2; Table 2.

Overall, these findings suggest that there was a 17% decrease in depression symptoms, 12% decrease in stress, and 16% increase in well-being in the awe intervention group (see ***%*** ∆ in Table 2; and Table S2 in supplemental material). These data provide evidence for the efficacy of an awe intervention, delivered virtually, in improving stress, depression symptoms, and well-being in people suffering from long COVID. The effect sizes between groups ranged from medium to large (*d* = 0.78–0.96), providing robust evidence for this awe intervention.

## Discussion

Experiences of awe are proposed to promote greater physical and psychological health^1,18,36^. We add to this literature by examining the effects of an awe intervention within a randomized clinical trial on the psychological health—stress, anxiety, depression, and well-being— of long COVID patients. This work documents the first RCT evidence on the effects of awe on psychological health on a unique patient population.

Our novel awe intervention promoted feelings of awe, more than the control group. This is consistent with other awe practices, including awe walks, awe narratives, nature immersions, among others^1^. In the examination of changes from baseline (T1) to post-intervention (T2), we found that the awe intervention, compared to control, promoted improvements in psychological health outcomes. Namely, those in the awe intervention, compared to control, showed decreases in reports of stress. This is in keeping with past work suggesting that experiences of awe reduce the subjective and physiological experience of stress^17,23^. Our new evidence, with a unique sample population, suggests that finding awe in everyday life— whether inside our home, our garden, or at a local park—can help reduce the impact of ongoing stressful experiences, such as long COVID.

The awe intervention also improved depression symptoms and well-being. Depression is one of the psychological health outcomes most impacted by the COVID-19 pandemic, with reports suggesting up to a 25% rise in the prevalence of depression worldwide^2^, and this was likely prolonged for people suffering from long COVID^7–9^. Our data provides evidence that individuals in the awe intervention, compared to the control group, exhibited significant improvements in symptoms of depression—up to a 17% decrease in depressive symptoms for the intervention group compared to a negligible 2% increase for those in the control group. This study is the first RCT to show the effects of awe on depression symptoms.

Consistent with work on the study of well-being^17,23,24,26,27^, we found that those in the awe intervention, compared to control, showed greater improvements in well-being—up to 16% increases in well-being for the intervention group. These findings demonstrate the efficacy of our novel intervention in promoting reductions in depression symptoms and improvements in well-being for people dealing with the long-term consequences of the COVID-19 pandemic.

This research builds on past work, suggesting that experiences of awe improve stress and well-being outcomes^17^, with a unique sample population. Notably, this work provides new evidence suggesting that a brief awe intervention can promote reductions in depressive symptoms. Findings from this study are robust, with effect sizes ranging from medium to large, *d* = 0.78 and 0.96 for depression and well-being, respectively. We did not find significant differences between the intervention and control group in anxiety symptoms. The null anxiety results might suggest that we are underpowered to test such effect. However, it could also suggest that when dealing with a chronic illness, such as long COVID, feelings of worry and anxiety can be more pervasive. Further work, with well-powered studies, is needed to examine these possibilities.

Our work contributes to a burgeoning science suggesting that positive emotions, such as awe, can be leveraged in interventions to improve physical and psychological health outcomes in clinical and non-clinical populations^12–15^. In keeping with other awe practices and interventions such as awe reflections^17^, nature outings^24^ and awe-walks^16^, among others in non-clinical populations^1^, this work suggests that finding brief moments of awe can lead to salutary effects. This work also adds to current efforts aimed at understanding and treating long COVID (e.g., the RECOVER NIH initiative^37^) by providing promising evidence that a brief awe intervention can promote greater psychological health in this critical population.

*Limitations and Implications for Future Work*. Future studies can improve on the limitations of this investigation. Given the sensitive nature and unpredictability of the COVID-19 pandemic, we recruited as many participants as possible within a short period of time. However, our sample sizes are relatively small (but adequate for simple group comparisons). Future work implementing this intervention can recruit larger samples and examine more complex models that can explore questions such as for whom the intervention was more effective. Although there was substantial attrition, there were no significant differences between those that dropped out and those that completed the intervention. Future studies can also assess outcomes of interest immediately after the intervention (e.g., a few days later as opposed to a month later), which could help reduce attrition rates. Moreover, since this intervention is the first trial of its kind, additional studies will be needed to replicate these results. In particular, future research can replicate this work with an active-control group, as opposed to a wait-list control as we did here, which would help reduce demand characteristics and possibilities of placebo effects. The large effect sizes found in this study are encouraging for the potential generalization of these findings and positions awe as a promising avenue to improve psychological health in chronic clinical conditions like long COVID.

## Electronic supplementary material

Below is the link to the electronic supplementary material.

Supplementary Material 1

## Data Availability

The data associated with this manuscript are available upon request to the corresponding authors.
