# Supplementary material for: Awe reduces depressive symptoms and improves well-being in a randomized-controlled clinical trial
Source: Sci Rep. 2025 May 12;15:16453. doi: 10.1038/s41598-025-96555-w (PMC12069556; doi:10.1038/s41598-025-96555-w)
Supplement: Supplementary file 1 — Supplementary Material 1 [file 41598_2025_96555_MOESM1_ESM.pdf]

Supplemental Material

Awe Reduces Depressive Symptoms and Improves Well-being in a Randomized-controlled Clinical Trial

María Monroy,* Michael Amster, Jake Eagle, Felicia K. Zerwas, Dacher Keltner, & Javier E. López*

* Corresponding Authors: m.monroy@yale.edu, drjlopez@ucdavis.edu

Section 1. Eligibility Screening Questions

To be eligible to participate in the study, interested participants had to meet a long COVID criteria based on the CDC.[1] This included having had a COVID infection, a documented positive test more than three months prior to recruitment, and currently having at least one of the known long COVID symptoms listed below.[1] For this final question interested participants completed the following question in yes or no format:

Are you **feeling unwell** and having **one or more** of the following symptoms since your positive COVID test?

- Coughing or feeling short of breath
- Loss of smell or change in taste
- Fever

- Body aches, headaches, chest pain, or stomach pain
- Brain fog (feeling like you can't think clearly)
- Having trouble sleeping
- Feeling very tired
- Mood changes

Interested participants were ineligible to participate if they were in the ICU, on a respirator, or their doctor performed any invasive heart (heart catheterization) or lung tests (bronchoscopy).

Early in recruitment, for a short period of time, people were also ineligible if they were on any treatment specific for their COVID symptoms (e.g., inhalers, blood thinners, heart medications). This is noted as "Early exclusion" (total 21) in Figure 1 in the main text. All exclusion changes were updated in the Clinical Trial registration.

Section 2: Additional Details About Procedures

The study design was a single-blind, waitlist-control, randomized clinical trial (RCT). All interested participants went through a common, 100% decentralized pathway for eligibility qualification, self-consent, enrollment, and questionnaire via the Qualtrics online platform. Entry point to this pathway was the UC Davis Health-supported StudyPages website (a portal facilitated by Yuzu Labs PBC, San Jose, CA, a HIPAA-compliant clinical research recruitment and engagement platform for online research conduct).[2] The website included basic information about the study and consent forms for self-consenting downstream of the eligibility screening survey and upstream from randomization.

Simple randomization was done (by MM) via a random number generator, in which patients were randomized into one of two groups: the intervention group and the waitlist-control group (see Figure 1 in the main text for enrollment flowchart). Participants were blinded to their group assignment.

In the current paper, we focus on measurements at two-time points: the pre-intervention data (T1) and the post-intervention data (T2; i.e., the month follow-up). At T2 only Group 1, the 'intervention group,' had received the intervention. This provides the cleanest comparison between the intervention group (Group 1) and the wait-list control group (Group 2). Additional procedures and assessments, not analyzed in this paper, included the following. Once the intervention started, for each group irrespective of intervention start date (see Figure S1), participants received a link to online diaries every Friday at 5pm for three weeks and every month for 3 months. Group 2 completed an additional monthly diary, see Figure S1 (Survey #5). Each diary began with Likert-type questions that prompted participants to report on their emotions, thoughts, and experiences during the past week or month.

Figure S1

Randomized Clinical Trial (RCT) Design

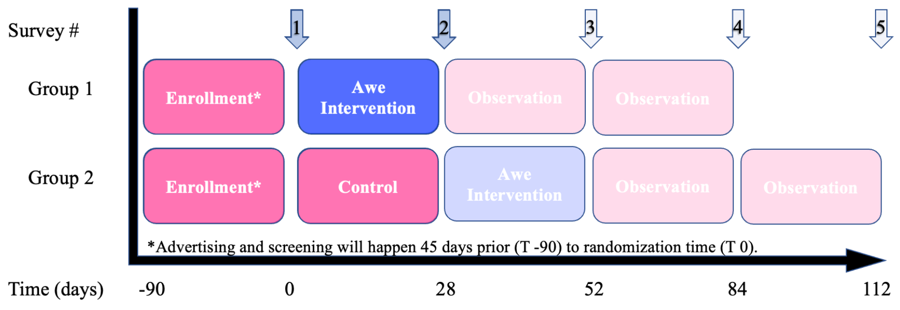

Note. Intervention design. In the main text we use T1 and T2 that depicts measurement number (i.e., Survey #)

Online Zoom Sessions

The first online session consisted of an overview of the study, an overview of the science of awe, and the core intervention component that entailed a discussion of how to find awe in the ordinary (by paying attention to the environment of daily living, slowing down, and expanding on those awe moments). Most information was presented during the first session and reviewed in the following three sessions. Given that the focus of the intervention was on ordinary awe, discussions entailed diverse types of awe, everyday awe, and that finding awe does not require extraordinary events. Examples were given of finding awe inside one's own home (e.g., by listening to music, watching droplets of water in slow motion), in the garden, or at a local park. The key point was the intervention component of finding awe in the ordinary (see main text).

Section 3. Adherence and Attrition

Given the attrition of the study (see Figure 1 in the main text), we compared differences in baseline measures (T1) of those who adhered to the intervention (completed T2 measures; $N = 68$) and those who dropped out ($N = 50$). We found no significant differences in all psychological health outcomes: stress ($p = 0.35$), anxiety ($p = 0.28$), depression ($p = 0.66$), or well-being ($p = 0.88$). We also did not find any differences in demographics (ps ≥ 0.29), except for age ($p = 0.02$). Those who continued with the intervention and completed T2 measures were slightly older ($M_{age} = 54.09$, $SD = 13.32$), than those who dropped out ($M_{age} = 48.16$, $SD = 12.67$).

Section 4. Stress Measure

To assess stress, we used five items of the perceived stress scale (PSS), [3] in which participants reported how often they felt or thought in certain ways during the last month (four weeks) on a scale from 1 (*never*) to 5 (*very often*): (1) *how often have you felt nervous and stressed?* (2) *how often have you felt that you were unable to control the important things in your life? (3) how often have you felt confident about your ability to handle your personal problems?* (4) *how often have you felt that things were going your way?* (5) *how often have you felt difficulties were piling up so high that you could not overcome them?* All items were aggregated into a composite (α_1 =.86, α_2 =.81). However, due to an item overlap ("*nervous")* with the generalized anxiety disorder scale (GAD), [4] we excluded item 1 from all analyses. All results are similar using 4 (PSS-4) or 5 items (PSS-5), and not statistically different (*p*s > 0.20). See Table S1 for descriptives for the PSS-5.

To replicate the main analyses examining the efficacy of the intervention on stress changes, we found similar results using the PSS-4 (see main text) and PSS-5: $\Delta M_{Awe} = -0.39$, $SE = 0.09$; $\Delta M_{Control} = 0.07$, $SE = 0.09$; $t(65.50) = - 3.64$, $p < 0.001$; Cohens' $d = 0.89$.

Table S1

Descriptive Statistics for T1 and T2 and Relative Changes of Stress (PSS-5)

		**Awe Intervention**			**Control**		
**Outcome Measure**	***Time***	***n***	***M (SE)***	***% Δ***	***n***	***M (SE)***	***% Δ***
Stress	T1	30	3.45 (0.14)		38	3.48 (0.13)	
	T2	30	3.06 (0.13)	- 11%	38	3.56 (0.11)	+ 2%

Section 5. Ancillary Discussion about Within-Group Results

Table S2, a replica of Table 2 from the main text with additional information, illustrates psychological health averages assessed at baseline or pre-intervention (T1) and at post-intervention (T2). As evident on Table S2, there is a clear pattern of results that are consistent with our primary findings: participants in the intervention group showed significant improvements in psychological health, while there were insignificant changes for those in the control group.

Table S2

		Awe Intervention				Control			
**Outcome Measure**	***Time***	***n***	***M (SE)***	***t***	***p***	***n***	***M (SE)***	***t***	***p***
Stress	T1	30	3.28 (0.15)			38	3.31 (0.14)		
	T2	30	2.90 (0.13)	4.00	< 0.001	38	3.39 (0.11)	- 0.83	0.410
Anxiety	T1	30	2.95 (0.15)			38	3.17 (0.18)		
	T2	29	2.65 (0.16)	2.32	0.028	38	3.08 (0.16)	0.85	0.401
Depression	T1	30	12.87 (0.94)			38	13.00 (0.89)		
	T2	29	10.62 (0.86)	3.99	< 0.001	38	13.21 (0.83)	-0.38	0.704
Well-being	T1	30	2.55 (0.18)			38	2.83 (0.16)		
	T2	30	2.95 (0.17)	-.3.31	0.002	38	2.60 (0.16)	2.10	0.042

Note. T1 denotes baseline/pre-intervention, and T2 denotes post-intervention. *M* denotes the mean and *SE* the standard error. Statistical significance of *t* tests between T1 and T2 within each group is also reported. Of note, the variation in *n* at T2 for the intervention group was due to missing data for 1 person.

References

1. CDC. Long COVID or Post-COVID Conditions. *The Centers for Disease Control and Prevention* https://www.cdc.gov/coronavirus/2019-ncov/long-term-effects/index.html (2023).
2. Sarraju, A. *et al.* Pandemic-proof recruitment and engagement in a fully decentralized trial in atrial fibrillation patients (DeTAP). *NPJ Digit Med* **5**, (2022).
3. Cohen, S., Kamarck, T. & Mermelstein, R. A Global Measure of Perceived Stress. *J Health Soc Behav* **24**, 385–396 (1983).
4. Spitzer, R. L., Kroenke, K., Williams, J. B. W. & Löwe, B. A Brief Measure for Assessing Generalized Anxiety Disorder. *Arch Intern Med* **166**, 1092–1097 (2006).
